# Modeling the Fundamental Viscoelastic Properties of Polylactic Acid (PLA) and PLA/Nanocomposites in a Unified Manner

**DOI:** 10.3390/nano14131116

**Published:** 2024-06-28

**Authors:** Evagelia Kontou, Ilias Charitos, Anastasios Drougkas

**Affiliations:** 1 Mechanics Department, School of Applied Mathematical and Physical Sciences, National Technical University of Athens, Iroon Polytechniou 9, Zografou, 15780 Athens, Greece; haritos42@hotmail.com; 2 Department of Civil and Environmental Engineering, Universitat Politècnica de Catalunya, Jordi Girona 1-3, 08034 Barcelona, Spain; anastasios.drougkas@upc.edu

**Keywords:** mechanical properties, relaxation, viscoelastic properties, retardation spectrum, modeling

## Abstract

The description of various loading types within the frame of viscoelasticity, such as creep–recovery and stress relaxation in a wide time scale, by means of the same model and similar model parameters is always an interesting topic. In the present work, a viscoelastic model that was analyzed in previous works has been utilized to describe the main standard loading types of viscoelasticity with the same set of model parameters. The relaxation function of this model includes a distribution function followed by the energy barriers that need to be overcome by the molecular domains when a stress field is applied. This distribution function attains a decisive role in the analysis and it was shown that it can be determined on the basis of the loss modulus master curve experimental results. Thereafter, requiring no additional parameters, the creep compliance, the relaxation modulus of poly-lactic acid (PLA) in a wide time scale, as well as creep–recovery at various stresses could be predicted. It was also found that by employing the distribution function associated with the PLA matrix, the creep–recovery experimental data of PLA/hybrid nanocomposites could subsequently be predicted. Therefore, the proposed analysis was shown to be a useful method to predict the material’s viscoelastic response.

## 1. Introduction

Due to the extensive use of polymers as parts of load-bearing structures, the description/prediction of their viscoelastic response, in terms of creep, relaxation, and monotonic loading is of great significance. Numerous works dealing with experiments and modeling the viscoelasticity of polymer and polymer composites have been performed so far, whereas, particularly, the creep response has been the concentrated the interest of many experimental and theoretical works [1,2,3,4,5,6,7,8,9,10]. For instance, the creep and creep–recovery response of polyolefin–rubber nanocomposites was modeled by the four-parameter Burger’s model [11]. Numerical modeling has been also employed, providing numerical solutions consistent with the experimental results. With the same trend, creep and recovery behaviors of continuous fiber-reinforced composites [12] and polyvinyl chloride [13] were experimentally studied and simulated with models. In addition, modeling the effects of nano-sized calcium carbonate particles and applied stress on the nonlinear viscoelastic behavior of high-density polyethylene has been performed by short-term creep experiments [14]. Polylactic acid (PLA), a well-known biodegradable polymer produced from renewable resources, is developed as a maintainable material in biomedical engineering, drug delivery, and as a scaffold in tissue engineering [15], while recently drawing attention in 3D printing applications [16]. Therefore, the mechanical and rheological properties of pristine PLA, or as a part of polymeric blends [17,18], and of PLA/nanocomposites have been the subject of numerous works [19,20,21,22].

With this trend, the effect of the microstructure on the mechanical properties and the energy dissipation capacity of the viscoelastic materials are matters of importance. In [23], a mathematical model in view of the effects of molecular chain–network configurations and temperature on the mechanical properties of viscoelastic materials was developed and analyzed.

Modeling of viscoelasticity by employing fractional derivative viscoelastic models has also extensively examined [24,25,26,27,28,29,30,31]. Fractional calculus has been proven to be an alternative for the analysis of both linear viscoelastic functions and dynamic responses. Within this context, a modified fractional Kelvin model taking into account the temperature and frequency impacts was proposed [32] and confirmed by dynamic mechanical analysis results for a nitrile rubber viscoelastic material. These material types are commonly used in the seismic vibration suppression of building structures.

However, it is a matter of great importance to analyze this response within the frame of a unified model, given that the majority of related works are referring to each loading type separately. Within this context, the behavior of the polymer composite, dependent on the stress history, was modeled using the Schapery single integral equation and the calculations were performed by a method based on Prony’s series [33]. The theoretical results were compared with experiments performed on polymer composite materials under creep–recovery, relaxation and ramp loading. In addition, in a work by Hasan and Boyce [34], a model is presented that can describe, in a unified way, the experimental results of strain rate compression tests at different temperatures and creep tests at various stress levels and temperatures. With this trend, in [35], the main features of inelastic mechanical behavior of glassy state were studied theoretically and experimentally in terms of tensile stress–strain and tensile creep experiments. The viscoelastic and viscoplastic response were adequately described within the frame of the same model. Successful modeling of the dynamic moduli was achieved in [28], and further validation was performed using creep and relaxation experimental data. A good approximation with experimental data was acquired, while deviations were obtained at longer times. In previous works by Drozdov et al. [36,37], the viscoelastic response in terms of stress–strain creep/creep failure and stress relaxation was successfully analyzed in the nonlinear viscoelastic and viscoplastic regime. A time-dependent constitutive equation was derived, employing a kinematic procedure regarding the attachment and detachment of active molecular chains in localized regions of the polymeric structure. 

Within this context, the main scope of the present work is to analyze theoretically, in a unified manner, the main standard loading types of viscoelasticity, such as creep–recovery and stress relaxation for a polymeric material, namely, PLA. It was proved that each loading type could be modeled within a similar theoretical frame, employing the same set of model parameters, which have physical significance. The analysis was performed in the linear viscoelastic domain, and creep as well as stress relaxation behaviors were adequately described in a wide time scale, along with creep and creep–recovery at various stress levels. Furthermore, to study the stress–strain response of PLA in monotonic loading, the employed analysis was supplemented by a kinematic formulation that separates the strain into viscoelastic and viscoplastic parts, and thereafter the entire stress–strain curve could be modeled.

Especially, the model’s capability to describe long-term creep and stress relaxation, as well as creep–recovery strain with the same set of parameters, unlike the majority of relative works, is an important contribution. It is worth mentioning that usually creep–recovery strain is simulated by conventional viscoelastic models employing different sets of parameters for creep and recovery [13,15].

Given that the creep response of polymer nanocomposites, especially as members of structures, attracted the intense interest of numerous researchers, the present model was further utilized. It was shown that the distribution function of PLA, which is actually a model parameter, can be employed to predict the creep–recovery experimental data of a series of PLA/hybrid nanocomposites at various nanofiller loadings. This is an encouraging finding, demonstrating that the present analysis can be an effective tool to predict the polymer’s/nanocomposites’ viscoelastic response.

## 2. Constitutive Analysis

The viscoelastic model, employed in the present work, has been developed in previous works [37,38,39]. In what follows, the model’s capability of capturing some main aspects of polymer’s viscoelasticity requiring the same set of model parameters will be shown.

The model under consideration includes a time-dependent constitutive equation, which was derived initially by Drozdov [36], and it is based on a special kinematic consideration of the rearrangement rate (attachment and detachment) of localized meso-regions of the molecular structure, arising from the Tanaka and Edwards transient model [39]. The attempt rate of these molecular motions is dependent on a distribution function, which is followed by the energy barriers that the molecular domains need to overcome upon the imposition of strain. 

On the basis of these assumptions, it was shown that the stress σ(t) and strain ε(t) for a uniaxial tensile experiment at a constant temperature are related as follows:

(1) $$\begin{array}{l} \sigma \left(t\right)=\mu_{0}\;\varepsilon \left(t\right)+\mu \left[\varepsilon \left(t\right)-\int_{0}^{\infty }\Gamma \left(u\right)\;p\left(u\right)\;\mathrm{du}\;\int_{0}^{t}\exp\left(-\Gamma \left(u\right)\;\left(t-\tau \right)\right)\;\varepsilon \left(\tau \right)\;d\tau \right]\\ =\mu_{0}\;\varepsilon \left(t\right)+\mu \left[\varepsilon \left(t\right)-\int_{0}^{t}R\left(t-\tau \right)\;\varepsilon \left(\tau \right)\;d\tau \right] \end{array}$$

where μ is the elastic stiffness and μ_0_ the relaxed modulus. It is worth mentioning that the constitutive equation has the form of a memory integral, with t being the current time and τ the previous one. Function R(t), which attains the character of a relaxation function, is given as follows:

(2) $$R\left(t\right)=\int_{0}^{\infty }\Gamma \left(u\right)\;p\left(u\right)\;\exp\left(-\Gamma \left(u\right)t\right)\mathrm{du}\;$$

The quantity p(u) is the distribution function of energy levels of the meso-regions and Γ(u) expresses the ratio of the number of meso-regions that are rearranged per unit time to the total number of the meso-region ensemble [36], and is given as follows:

(3) $$\Gamma \left(u\right)=\gamma_{Τ}\exp\left[-u\right]$$

where γ_Τ_ is the attempt rate of the molecular rearrangements, considered dependent on temperature, and the term $\exp\left[-u\right]$ denotes the probability of a meso-region to cooperatively rearrange. It will be shown that p(u) plays a decisive role in the analysis.

Considering an imposed strain of type ε(t) = ε_0_ exp(iωt) with an amplitude ε_0_ and ω being the oscillation frequency related to a dynamic mechanical experiment, and following the constitutive Equation (1), the ratio σ(t)/ε(t) yields the complex modulus E*. After some calculations, which are analytically shown in [36], the complex modulus E*, as well as the real Ε′ and imaginary Ε″ part of E*, are given as follows:

(4) $$E^{*}=\mu_{0}+\mu \int_{0}^{\infty }\frac{i\omega }{\Gamma (u)+i\omega }p(u)\mathrm{du}\;\;E\prime (\omega )=\mu_{0}+\mu \int_{0}^{\infty }\frac{\omega^{2}}{{\left(\Gamma (u)\right)}^{2}{+\omega }^{2}}p(u)\;\mathrm{du}\;E\prime \prime \left(\omega \right)=\mu \overset{\infty }{\underset{0}{\int }}\frac{\Gamma \left(u\right)\omega }{{\left(\Gamma \left(u\right)\right)}^{2}+\omega^{2}}p\left(u\right)\mathrm{du}=\mu \overset{\infty }{\underset{0}{\int }}\frac{\Gamma \left(u\right)\omega }{{\left(\Gamma \left(u\right)\right)}^{2}+\omega^{2}}p\left(u\right)\mathrm{dlnu}$$

According to the analysis performed for the dielectric loss [40], similar equations are reported for the continuous relaxation time distribution function (RTDF) and its connection with the dielectric loss x″(ω). The analogy here is the connection between the distribution function p(u) and the loss modulus. Following Ref. [40], the solution for the p(u) in Equation (4) gives the following:

(5) $$\mu \;p[u]≅\frac{2}{\pi }\left[\;E\prime \prime (\ln\omega )-\frac{\pi^{2}d^{2}\;E\prime \prime (\ln\omega )}{8d{\left(\ln\omega \right)}^{2}}+\ldots \right]\;\mathrm{with}\;\omega =\Gamma (u)\;$$

Considering that the second term of the right hand in Equation (5) is negligible [38], we obtain the following approximation:

(6) $$p[u]≅\frac{2}{\mu \pi }\left[\;E\prime \prime \;(\ln\omega )\right]\;\mathrm{with}\;u=\ln\frac{\gamma_{T}}{\omega }$$

According to Equation (6), the distribution function p(u) can be calculated from the experimental data of the loss modulus E″(lnω). The calculation procedure is presented in the following paragraphs.

On the other hand, recalling the expressions of the dynamic moduli of the generalized Maxwell model [34] containing *N* elements, each with a modulus E*_j_* and relaxation/retardation time τ*_j_*, we have the following equation:

(7) $$\;E\prime (\omega )=E_{\mathrm{relax}}+\sum_{j=1}^{N}\frac{E_{j}\omega^{2}\tau_{j}^{2}}{{1+\omega }^{2}\tau_{j}^{2}}\;\mathrm{or}\;E\prime \prime (\omega )=\sum_{j=1}^{N}\frac{E_{j}{\omega \tau }_{j}}{{1+\omega }^{2}\tau_{j}^{2}}\;$$

where E′(ω), E″(ω) are the storage and loss modulus, E_relax_ is the relaxed modulus, and ω is the frequency. The analogy between Equations (4) and (7), demonstrates that the quantity Γ(u) = γ_Τ_ exp[−u] takes the place of the inverse of relaxation time τ*_j_*.

Furthermore, this analogy can be confirmed in view of the fact that the loss modulus E″(ω) or the loss compliance J″(ω) are related to the relaxation time spectrum H(τ), or the retardation time spectrum L(τ), which according to the Alfrey’s approximation, are given by the following equations [41,42]:

(8) $$H\left(\tau \right)=\frac{2}{\pi }{\left[\;E\prime \prime \left(\omega \right)\right]}_{1/\omega =\tau }\;\mathrm{and}\;L\left(\tau \right)=\frac{2}{\pi }{\left[\;J\prime \prime \left(\omega \right)\right]}_{1/\omega =\tau }$$

The physical significance of H(τ) and L(τ) is directly related to the distribution of relaxation/retardation times that characterizes the complex polymeric structure. As already mentioned, the scope of the present work is to explore and analyze the potential of this model to describe the main aspects of the viscoelastic behavior of the polymeric structure in a unified manner. Therefore, the experimental results for the storage and loss modulus, creep compliance, relaxation modulus, creep–recovery tests, and uniaxial tension of a well-known polymer, namely, polylactic acid (PLA), were employed. In the following paragraphs, different functions of viscoelasticity, such as creep compliance and the relaxation function along a wide time scale and creep–recovery strain at various stress levels, of a PLA material will be simulated by the model presented above with the same set of parameters. The study is further extended to the creep and creep–recovery behaviors of PLA/hybrid nanocomposites.

## 3. Materials and Methods

The polymeric material employed as a matrix is poly (lactic acid), designated as (PLA) under the commercial name Ingeo^TM^ Biopolymer 2003D, produced by NatureWorks LLC, Minneapolis, MN, USA, and was kindly supplied by the Greek Company M.Procos S.A, Athens, Greece. The selected grade 2003D has a density of 1.24 g/cm^3^ and a MFR index equal to 6 g/10 min, measured at 210 °C at a load of 2.16 kg, according to ASTM-D1238-65T. Prior to use, the material was formed in pellets and was dried at 45 °C for a minimum of 2 h in a desiccating dryer. Melt mixing of the PLA matrix material was performed with a Brabender mixer. The temperature was set at 160 °C and the rotation speed of the screws was 40 rpm. Thereafter, the material was compression molded at 160 °C using a thermo-press and a special mold of 1.5 mm thickness.

A similar melt mixing procedure has been followed for the preparation of PLA/hybrid nanocomposites based either on a mixture of graphene oxide (GO)/carbon nanotubes (CNTs) or on GO/carbon nanofibers (CNFs). PLA/GO/CNTs and PLA/GO/CNFs contain GO and CNT (or CNF) at equal ratios and at two different total nanofiller loadings, namely, 3.84, and 6.25 wt.%. The nanocomposites’ designations are shown in Table 1. A detailed description of the preparation procedure and the thermomechanical performance is presented in [21].

Dynamic mechanical analysis (DMA) was performed by a TA Q-800 Instrument (TA Instruments, New Castle, DE, USA). The mode of deformation applied was the single cantilever, and the mean dimensions of sample plaques were of 12.7 mm in width and 17.5 mm in length. The experiments were performed at a temperature range from 30 °C to 100 °C, with a heating rate of 3 °C/min. The material’s behavior regarding its temperature dependence was investigated by checking the force and angle phase variations, retaining the amplitude of oscillation constant. Four discrete frequencies, 1, 5, 10, and 20 Hz, were studied and the storage and loss moduli curves versus temperature were derived. 

Tensile measurements were carried out with an Instron 1121 type tester at room temperature, according to ASTM D638. The dumbbell-type specimens were of a gauge length of 20 mm, and the applied effective strain rate was equal to 4.17 × 10^−4^ s^−1^. A laser extensometer-type cross-scanner from Fiedler Optoelektronik GmbH, Lützen, Germany was used for the detailed deformation measurements.

Creep experiments were also performed with TA Q800 Instrument at a single cantilever beammode of deformation for a specific time period equal to 30 min at various temperatures varying from 37 to 80 °C, with an interval of 5 °C. A constant stress level of 10 MPa was applied. In addition, stress–relaxation experiments were performed for a time period equal to 30 min at a constant strain level, equal to 0.6%, and at various temperatures varying from 37 to 80 °C, with an interval of 5 °C. 

Independently, creep–recovery experiments for PLA at various stress levels, namely, 10, 15 and 20 MPa, were also performed at room temperature. This procedure included an abrupt imposition of a stress, which was kept constant with time. The creep time period was 20 min. Thereafter, the constant stress was removed and the recovery strain was recorded, with the creep–recovery duration being 40 min.

The research was extended to the creep and creep–recovery experiments of PLA/hybrid nanocomposites at room temperature and various stress levels. To have comparative results, the imposed stress was equal to 30, 45, and 60% of the corresponding yield stress of the material, as shown in Table 2.

## 4. Experimental Results

The tensile properties of PLA and PLA/hybrid nanocomposites, which were extensively examined in [21], are presented in Table 3.

**Figure 1:**
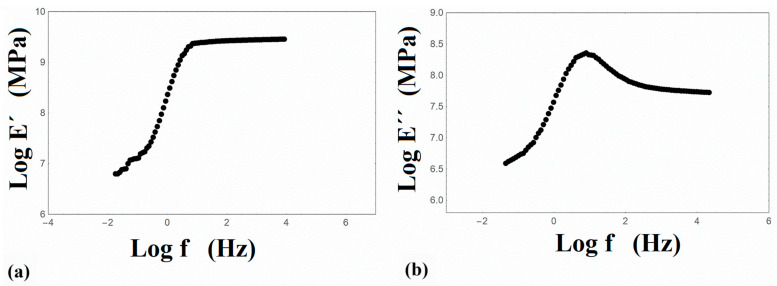
Master curves of (**a**) the storage E′ and (**b**) loss Ε″ moduli of PLA at a reference temperature of 60 °C.

The DMA performed at four discrete frequencies and the relaxation/creep experiments executed at various temperatures provide the necessary data for the application of the time–temperature superposition (TTS) principle [42]. Consequently, the master curves of the storage E′ and loss E″ moduli were derived and are plotted on a logarithmic frequency scale in Figure 1. It must be mentioned that three different experiments were performed with almost zero deviation in their results.

**Figure 2:**
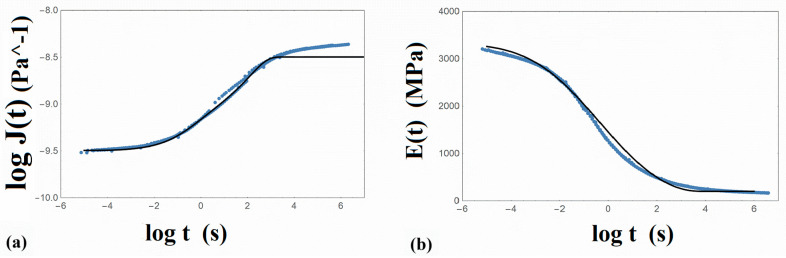
Master curves of the (**a**) logarithm of creep compliance J(t) and (**b**) the relaxation modulus E(t) of PLA at a reference temperature of 60 °C. Points: experimental data. Solid line: model simulation.

It is to be noted that the master curves of both dynamic moduli were constructed by the same shift factor values, which is an indication that the TTS principle is valid. The calculated values of the Williams–Landel–Ferry (WLF) equation constants are C_1_ = 19.4 and C_2_ = 148.3 (K). Additionally, the creep compliance J(t) (Figure 2a) and the relaxation modulus E(t) (Figure 2b) on a logarithmic time scale were acquired. All master curves were constructed at a reference temperature 60 °C, which is close to the glass transition temperature of PLA.

**Figure 3:**
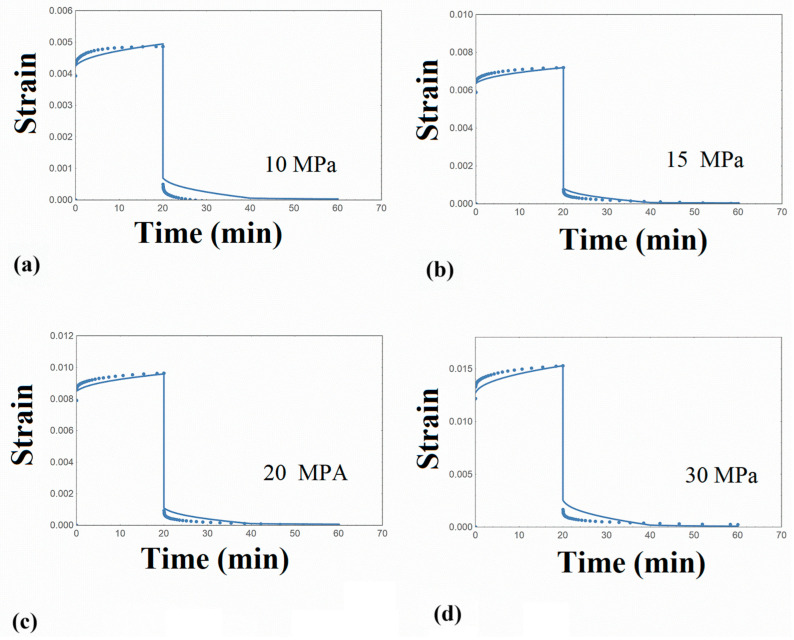
Creep–recovery strain curves of PLA at room temperature and various stress levels (**a**) 10 MPa (**b**) 15 MPa (**c**) 20 MPa (**d**) 30 MPa.Points: experimetal data. Solid line: model simulation.

**Figure 4:**
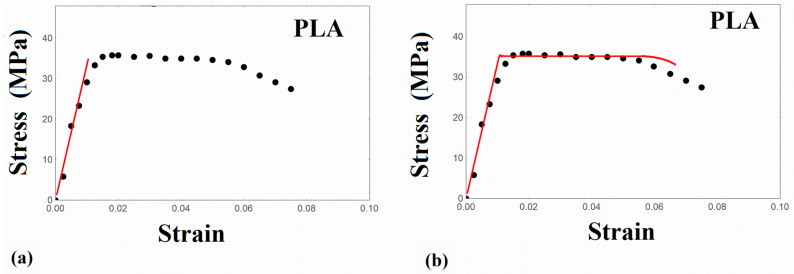
Tensile stress–strain curves of PLA at room temperature. Points: experimental data. (**a**) Solid line: linear viscoelastic model. (**b**) Solid line: viscoplastic model.

The creep–recovery experiments at room temperature are illustrated in Figure 3, and the tensile stress–strain curve of PLA is also depicted in Figure 4. It is observed that PLA exhibits a well-defined yield stress after the initial elastic/viscoelastic region, followed by a slight strain softening, and the stress is kept rather constant until failure. The strain at break is about 8%, revealing a rather brittle material.

## 5. Model Validation

In this section, the implementation of the above presented model for simulating the creep compliance, the relaxation function, and creep–recovery strain of the PLA polymer are shown. In addition, on the basis of the calculated distribution function p(u) for the PLA matrix, which is a decisive parameter for our analysis, the creep–recovery strain of PLA/hybrid nanocomposites will also be simulated.

**Figure 5:**
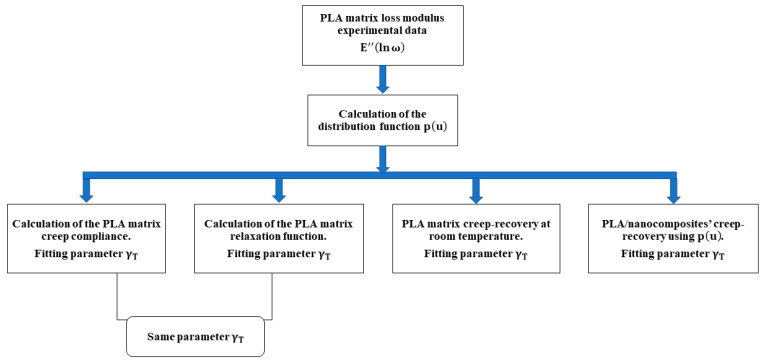
Flow diagram of model calculations.

The calculation procedure followed in the present work can be summarized in the flow diagram shown in Figure 5.

### 5.1. Calculation of the Distribution Function p(u)

The experimental data for the loss modulus master curve were transformed to a function through an interpolation procedure by the software Mathematica 11. By this procedure, which is a simple input within the frame of the software, introducing the experimental points of the loss modulus versus frequency, an approximate function p(u) is constructed. According to the interpolation procedure within the frame of the software employed, the function p(u) is strictly defined between two specific limits u_1_ and u_2_. Any calculation related to the function p(u) can only be performed between these limits. The specific formula of this function is not known, but it is possible to obtain its value at any point in the defined limits. Additionally, this function can be plotted and included in calculations such as integration. Hereafter, the function p(u) is normalized by division to the quantity:

(9) $$p_{0}=\int_{u_{1}}^{u_{2}}p(u)\mathrm{du}\;$$

**Figure 6:**
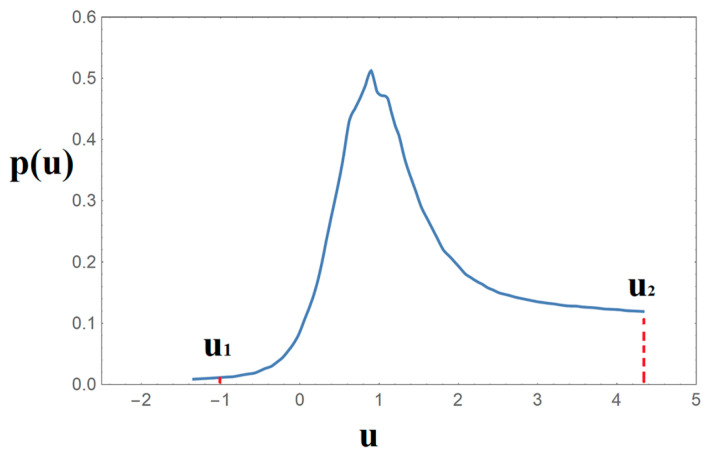
The distribution function p(u) as derived from the master curve of the loss modulus of PLA.

In this way, the normalized function p(u) attains the character of a distribution function. On the basis of the loss modulus experimental data, the obtained distribution function p(u) is finally illustrated in Figure 6. It is to be noted that the limits u_1_ and u_2_ may have negative values, meaning that these limits actually denote the height distance from a reference energy level, that the molecular domains have to overcome for the molecular rearrangements to occur.

### 5.2. Calculation of the Creep Compliance and Relaxation Modulus

The creep compliance function J(t) is expressed in terms of the dynamic compliance J″(ω) by the Fourier transform procedure using the following well-known equation [42]:

(10) $$J(t)=J_{g}+\frac{2}{\pi }\int_{-\infty }^{\infty }\;J\prime \prime (\omega )\;(1-\cos\omega t)\;\mathrm{dln}\omega$$

where J″(ω) is the loss compliance of the material, and J_g_ the glassy compliance, which is the reciprocal of the instantaneous modulus [42] and corresponds to the compliance value at the lower values of the time scale. Therefore, taking an approximate value for the elastic stiffness μ equal to 3000 MPa (Table 4), the glassy compliance J_g_ is equal to (1/3.0) 10^−9^ Pa^−1^. In Figure 2a, the logarithmic value of J_g_ is presented in the lowest time regime. On the other hand, the loss compliance is given as follows:

(11) $$J\prime \prime \;(\omega )=\frac{{E\prime \prime }^{2}\left(\omega \right)}{{\;E\prime \prime }^{2}{(\omega )+E\prime }^{2}\left(\omega \right)}$$

Following Equation (11), the loss compliance J″(ω) was evaluated by employing the simulated values of the dynamic moduli E′(ω) and Ε″(ω), expressed in Equation (4). Then, the creep compliance could be calculated by Equation (10) and presented in Figure 2a in comparison with the experimental data. From this plot, a satisfactory prediction of the creep compliance J(t) was achieved in the entire time range examined, i.e., from 10^−5.8^ s to 10^6^ s, with the exception of the long time scale beyond 10^3^ s, where it was not possible to predict the increasing slope of the experimental master curve of PLA. This response may be correlated with particular molecular motions that cannot be described by the model employed.

The relaxation modulus E(t) could be simulated by the constitutive Equation (1), and solved for stress at a constant strain ε_0_ = 0.6%. By setting ε(t) = ε_0_ in Equation (1), the relaxation modulus E(t) = σ(t)/ε_0_ is given as follows:

(12) $$\begin{array}{l} E\left(t\right){=\mu }_{0}+\mu \;\left[1-\int_{0}^{\infty }\Gamma \left(u\right)\;p\left(u\right)\;\mathrm{du}\;\int_{0}^{t}\exp\left(-\Gamma \left(u\right)\;\left(t-\tau \right)\right)d\tau \right]\\ {=\mu }_{0}+\;\mu \left[1-\int_{0}^{t}R\left(t-\tau \right)d\tau \right] \end{array}$$

The model-simulated relaxation modulus E(t) is presented in comparison with the relaxation modulus experimental master curve in Figure 2b. A very satisfactory prediction of the relaxation modulus was achieved. It is to be noted that all calculations were performed numerically by assuming small time steps, while the convergence was carefully controlled.

Apart from the calculated function p(u) derived from the loss modulus data, the model parameters required are Young’s modulus μ and the attempt rate γ_T_, and are shown in Table 4. Actually, the only fitting parameter is the attempt rate γ_T_, which was estimated to be equal to 25 s^−1^. The estimation of the parameter γ_T_ was performed using a trial and error method for both quantities, the creep compliance and the relaxation modulus. The selected γ_T_ value was the one offering the best approximation with the experimental data for both J(t) and E(t). Given that this is the only fitted parameter, its estimation can be achieved with good accuracy.

### 5.3. Calculation of the Creep–Recovery Strain of PLA

Τhe creep strain is calculated from the constitutive Equation (1) on the basis of the calculated distribution function p(u) by imposing a constant stress σ_0_, as follows:

(13) $$\;\varepsilon \left(t\right)=\frac{\sigma_{0}}{\mu }+\int_{0}^{t}R\left(t-\tau \right)\varepsilon \left(\tau \right)\;d\tau \;\;$$

**Figure 7:**
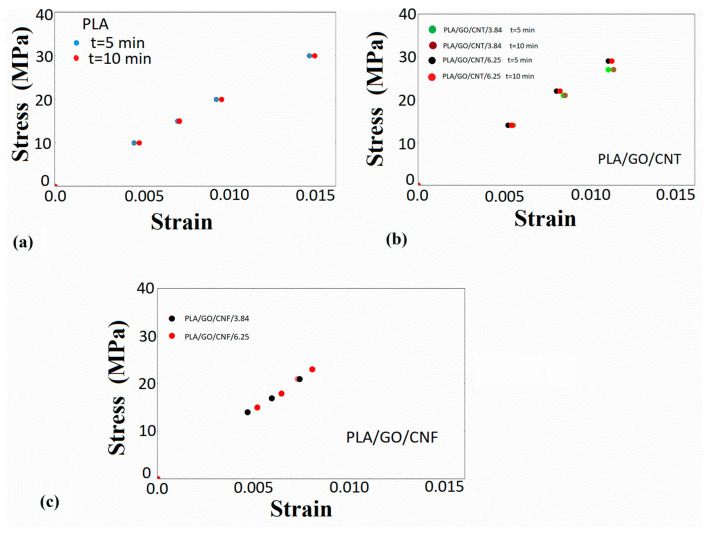
Creep isochrones of (**a**) PLA at t = 5, 10, and 20 min, (**b**) PLA/GO/CNT nanocomposites, and (**c**) PLA/GO/CNF nanocomposites.

The calculations were made using small time steps until a convergence could be achieved, while a long computing time was required. The simulated creep strain–creep recovery curves are shown in Figure 3, together with the experimental results for the various stress levels examined. On the basis of these experimental results, stress–strain diagrams for a specific time can be constructed, the so-called isochrones. The deviation of the linear trend in these plots denotes the onset of nonlinearity. By plotting the isochrones of the experimental creep strain results in Figure 7a, it can be observed that the creep stresses imposed are related to the linear viscoelastic response of PLA. 

In Figure 3, a quite satisfactory estimate was achieved between the experiment and model simulation. The model parameters required are Young’s modulus μ and the attempt rate γ_T_. As already mentioned, the creep–recovery experiments were performed at room temperature, and the distribution function was evaluated on the basis of the loss modulus master curve, which was constructed at a reference temperature equal to 60 °C. Therefore, for the calculation of the creep compliance and relaxation modulus, which were constructed at the same reference temperature, a single value of parameter γ_T_ was employed to simulate both the J(t) and E(t) master curves.

Regarding the creep–recovery experiments at room temperature, it is reasonable to consider that a different γ_T_ value than the one estimated for J(t) and E(t) is required, given that these master curves are referred to a temperature of 60 °C. To simulate the creep curves obtained at room temperature at the first stress level, we started the numerical calculations on the basis of Equation (13), taking into account Equations (2) and (3) where p(u) is known and contributes to the calculations. The only fitting parameter is γ_T_, and a value is assumed in order to perform the numerical calculations. We used a trial and error method, and when a good fit to the experimental creep strain was achieved with a specific γ_T_ value, we proceeded with the same procedure for the second stress level. Using a similar procedure and adopting the same γ_T_ value as the one for the first stress level, we could simulate with good accuracy the creep strain. Generally, it was found that the creep strain at the subsequent stress levels could be calculated with almost the same parameter value, performing a back analysis procedure. Based on these results, the attempt rate value at room temperature was found, on average, to be equal to 8.2 10^−4^ s^−1^. The temperature dependence of parameter γ_T_ may follow an Arrhenius or Vogel–Fulcher [41] formula, and in order to study this dependence, a set of experimental data at various temperatures would be necessary. What is important to be mentioned is that all creep–recovery curves (Figure 3) could be simulated with the same set of parameters, as shown in Table 4, in a quite satisfactory manner.

This is not the case for numerous previous studies, where a different set of parameters is required for the simulation of the creep–recovery stage at various stress levels [5,6,13,15,29,30,31]. More specifically, the creep strain is usually simulated by a well-known linear viscoelastic four-parameter model, whereas a different model is employed to describe the recovery strain, requiring additional parameters [11,12,13].

### 5.4. Modeling the Tensile Stress–Strain Curve

In the experimental tensile stress–strain curve of PLA in Figure 4, the initial elastic/viscoelastic region is observed, which is followed by yielding with the appearance of a stress peak, and thereafter a stress softening is obtained and finally the material is driven to failure. The tensile experimental data were simulated within the context of the present viscoelastic model. On the basis of the constitutive Equation (1), calculations were performed by employing the already calculated distribution function p(u) and the γ_T_ equal to the one utilized in the creep–recovery experiments at room temperature. Due to the linear character of the viscoelastic model under consideration, a single linear stress–strain curve is obtained and shown in Figure 4a. This curve is capable of capturing only the initial elastic/viscoelastic part of the materials’ response.

To proceed to a detailed simulation of the stress–strain curve, an appropriate separation of the total strain into viscoelastic and plastic part is required. A kinematic formulation of the rate of plastic deformation [43], utilized in our previous works [35,44], was proven to successfully capture the viscoplastic response of the materials.

According to this formulation, for axial loading, the rate of the elastic stretch ratio $\frac{{d\lambda }_{\mathrm{el}}}{\mathrm{dt}}$ is given as follows [35,44]:

(14) $$\frac{\frac{{d\lambda }_{\mathrm{el}}}{\mathrm{dt}}}{\lambda_{\mathrm{el}}}=\left[\frac{1+\frac{1-2\nu }{2(1+\nu )}\left(\frac{\lambda_{\mathrm{el}}^{3}-1}{\lambda_{\mathrm{el}}}\right)}{1+\frac{1-2\nu }{6(1+\nu )}\left(\frac{{5\lambda }_{\mathrm{el}}^{3}-2}{\lambda_{\mathrm{el}}}\right)}\right]\;\left[\frac{r}{\lambda_{\mathrm{el}}}-\frac{G}{18}\left(\frac{\lambda_{\mathrm{el}}^{3}}{\lambda_{\mathrm{el}}}\right){\;(4\lambda }_{\mathrm{el}}^{3}+2)\right]$$

where r is the imposed strain rate, $\lambda_{\mathrm{el}}$ = 1 + ε_el_ with ε_el_ as the elastic strain, λ_el_ is the elastic stretch ratio in the loading axis, and ν is Poisson’s ratio. G is the functional form of the rate of plastic deformation, which has been introduced in previous works [33,42] and has the form of a Gaussian distribution with a standard deviation s equal to 0.001 and a mean value ε_y_ equal to the yield strain:

(15) $$G=\frac{r}{{\lambda \varepsilon }_{y}}\int_{0}^{\varepsilon }\frac{1}{s\sqrt{\pi }}\exp\left[-0.5{\left(\frac{{\varepsilon -\varepsilon }_{y}}{s}\right)}^{2}\right]\;d\varepsilon$$

where λ is the total stretch ratio in the loading axis. Combining Equations (14) and (15) with the constitutive Equation (1) and performing numerical calculations, the experimental results of Figure 4 were simulated, exhibiting a satisfactory approximation with the experimental stress–strain curve, as illustrated in Figure 4b. The value of ε_y_ was equal to 0.008. With the present model, all the details of the stress–strain curve could be described in a satisfactory estimate. In Table 4, the model parameters for each loading type are presented, and it is worth mentioning that a limited number of parameters is necessary to describe different loading types of viscoelasticity.

### 5.5. Model Simulation of the Creep–Recovery Strain of PLA/Hybrid Nanocomposites

**Figure 8:**
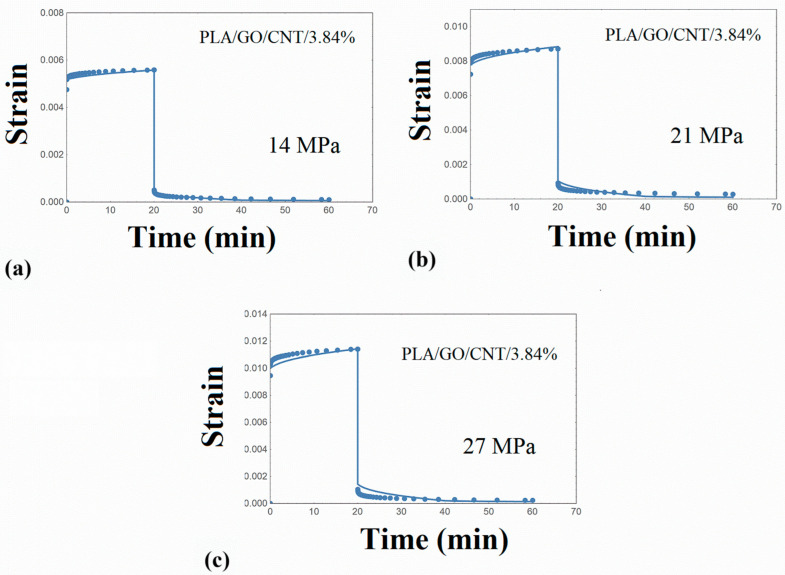
Creep–recovery strain for PLA/GO/CNT/3.84% at various stress levels (**a**) 14 MPa (**b**) 21 MPa (**c**) 27 MPa. Points: experimental data. Solid line: model simulation.

**Figure 9:**
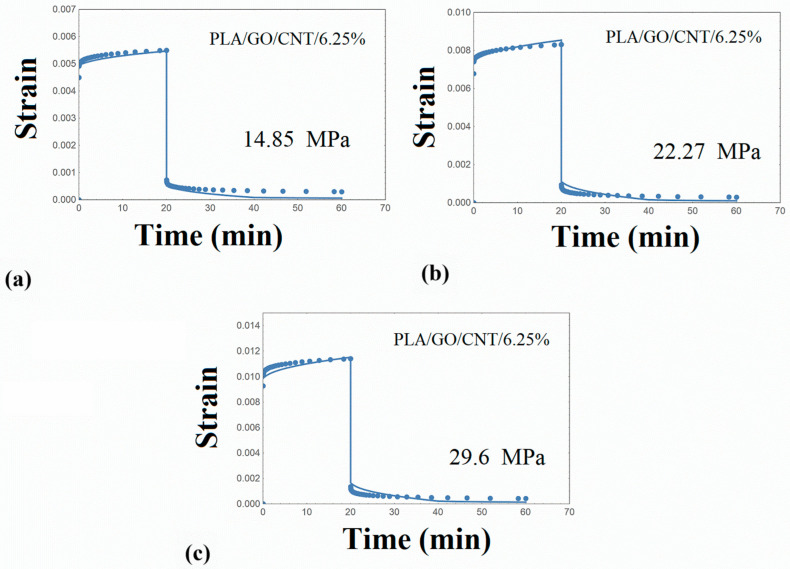
Creep–recovery strain for PLA/GO/CNT/6.25% at various stress levels: (**a**) 14.85 MPa (**b**) 22.27 MPa (**c**) 29.6 MPa. Points: experimental data. Solid line: model simulation.

**Figure 10:**
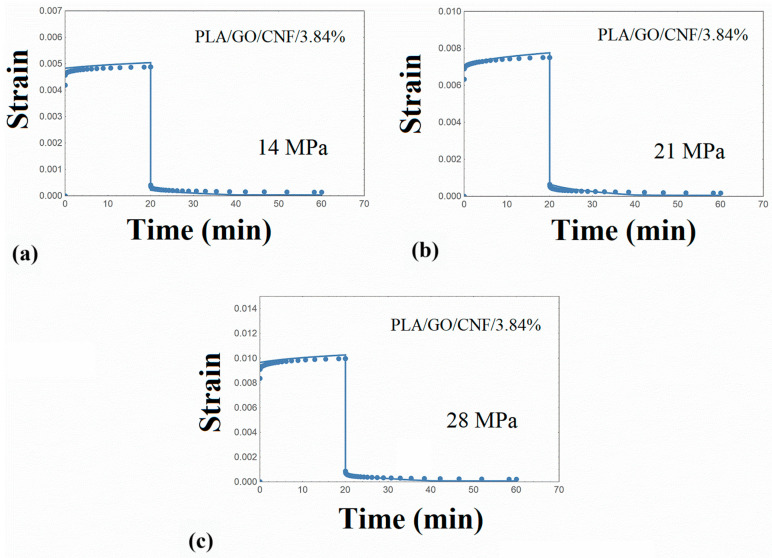
Creep–recovery strain for PLA/GO/CNF/3.84% at various stress levels (**a**) 14 MPa (**b**) 21 MPa (**c**) 28 MPa. Points: experimental data. Solid line: model simulation.

**Figure 11:**
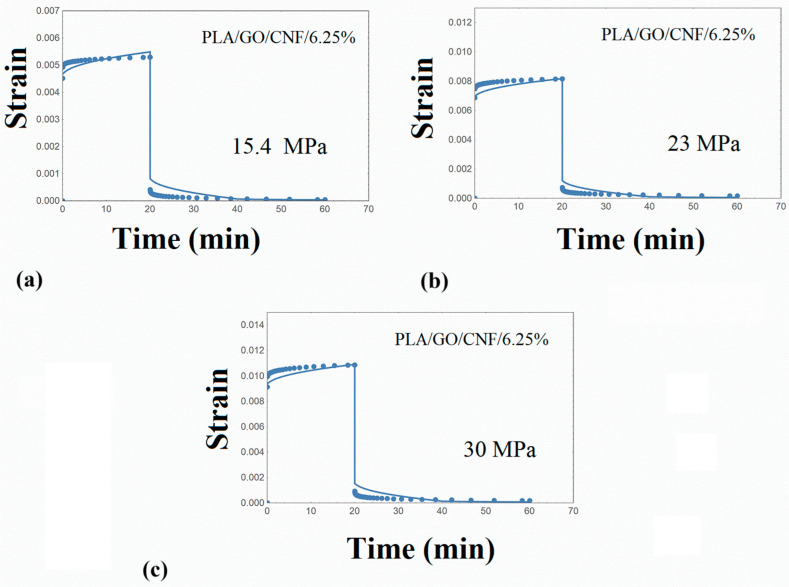
Creep–recovery strain for PLA/GO/CNF/6.25% at various stress levels (**a**) 15.4 MPa (**b**) 23 MPa (**c**) 30 MPa. Points: experimental data. Solid line: model simulation.

The creep–recovery plots of the PLA/hybrid nanocomposites at various stresses are illustrated in Figure 8, Figure 9, Figure 10 and Figure 11, while the corresponding isochrone plots are shown in Figure 7b,c. From these plots, it is extracted that the nonlinear viscoelasticity threshold is about 22 MPa for PLA/nanocomposites.

Performing similar numerical calculations as in the previous paragraphs, the creep strain curves were adequately predicted, as shown in Figure 8, Figure 9, Figure 10 and Figure 11. It is to be noted that the distribution function p(u) estimated for the PLA matrix was employed in the simulation procedure of the PLA/hybrid nanocomposites. Therefore, the set of required parameters is limited to the elastic stiffness μ and parameter γ_T,_ and this is an interesting finding and can be used in future related works. The model parameters required for the PLA/nanocomposites are shown in Table 5.

It can be seen from Table 5 that the attempt rate value is the same for every type of PLA/hybrid nanocomposite. It has the same value for PLA/GO/CNT/nanocomposites at the two different nanofiller loadings and a different value for the PLA/GO/CNF nanocomposites at the two different nanofiller loadings. It is to be noted that this value is constant for all the stress levels examined and the subsequent recovery stage.

The results of these simulations are very encouraging, revealing the potential of the employed model to describe and predict the viscoelastic response of high-performance PLA/carbonaceous hybrid nanocomposites with the distribution function p(u) of PLA matrix, and requiring thereafter a limited number of parameters.

## 6. Conclusions

In the present work, a model is employed to describe/predict the main features of the viscoelastic behavior of polymers in a unified manner. It was found that standard loadings of viscoelasticity performed at the same temperature are described by the same parameter values, unlike the majority of comparable studies. By this model, which was introduced in previous works, a time-dependent constitutive equation is derived that has the type of a memory integral. The polymeric structure is assumed to be an ensemble of molecular localized regions that participate in molecular rearrangements under the imposition of a stress/strain field. The distribution function, which is obeyed by the energy barriers that the domains have to overcome, is shown to play a key role, since it is related to the distribution of the material’s relaxation/retardation time spectrum. This function can be determined from the loss modulus versus frequency experimental results, and thereafter the creep/recovery data, the compliance, and relaxation modulus in a wide time scale for a selected polymeric material, namely, PLA, can be predicted. In addition, the linear part of the tensile stress–strain curve can be simulated. For the viscoplastic region’s simulation, the separation of the total strain into viscoelastic and viscoplastic parts by a proper kinematic formulation is necessary.

It is of great importance and crucial for a number of applications to predict the long-term creep strain and the rheological response of polymers in general; therefore, the findings of the present research are encouraging, revealing the model’s potential to simulate fundamental viscoelastic properties. Particularly, the model’s capability to capture the long-term creep compliance and relaxation modulus, as well as creep–recovery strain at various stresses, with the same set of parameter values constitutes an important contribution. It was also found that by employing the distribution function defined for the PLA matrix, it is possible to simulate the creep–recovery strain of a series of PLA/hybrid nanocomposites, thus needing a limited number of model parameters for analyzing high-performance materials.

## Figures and Tables

**Table 1:** PLA/hybrid nanocomposites investigated and related designations.

Material	Total Nanofiller Weight Fraction (%)
PLA	-
PLA/GO/CNT/3.84%	3.84
PLA/GO/CNT/6.25%	6.25
PLA/GO/CNF/3.84%	3.84
PLA/GO/CNF/6.25%	6.25

**Table 2:** Creep stress levels imposed for all materials examined.

Material	Yield Stress (MPa)	Creep Stress (MPa)
PLA	34.4	10.0, 15.0, 20.0, 30.0
PLA/GO/CNT/3.84%	46.7	14.0, 21.0, 27.0
PLA/GO/CNT/6.25%	49.5	14.85, 22.27, 29.6
PLA/GO/CNF/3.84%	47.4	14.0, 21.0, 28.0
PLA/GO/CNF/6.25%	51.4	15.4, 23.0, 30.0

**Table 3:** Tensile results of PLA and PLA/hybrid nanocomposites (Ref. [21]).

Material	Young’s Modulus (MPa)	Yield Stress (MPa)	Yield Strain (%)	Tensile Strength (MPa)
PLA	3064	34.4	1.52	27.0
PLA/GO/CNT/3.84%	3218	40.2	1.48	29.5
PLA/GO/CNT/6.25%	4265	40.4	1.43	25.9
PLA/GO/CNF/3.84%	3460	38.5	1.28	28.9
PLA/GO/CNF/6.25%	3796	-	-	43.9

**Table 4:** Model parameter values for PLA.

Experiment Type	γ_T_ (s^−1^)	μ (MPa)
Creep compliance T_ref_ = 60 °C	25	3000
Relaxation modulus T_ref_ = 60 °C	25	3000
Creep–recovery at room temperature	8.2 × 10^−4^	3000
Tension at room temperature	8.2 × 10^−4^	3000

**Table 5:** Model parameters for the creep–recovery of the PLA/hybrid nanocomposites examined.

Material	γ_T_ (s^−1^)	μ (MPa)
PLA/GO/CNT/3.84%	6.14 × 10^−^^6^	3000
PLA/GO/CNT/6.25%	6.14 × 10^−^^6^	3300
PLA/GO/CNF/3.84%	2.26 × 10^−^^6^	3000
PLA/GO/CNF/6.25%	2.26 × 10^−^^6^	3200

## Data Availability

Dataset available on request from the authors.
